# Systemic Lupus Erythematosus and Antiphospholipid Syndrome Accompanied by Mixed-Type Autoimmune Hemolytic Anemia

**DOI:** 10.1155/2023/4963196

**Published:** 2023-09-19

**Authors:** Eiji Suzuki, Takashi Kanno, Yurie Saito, Takuro Shimbo

**Affiliations:** ^1^Department of Rheumatology, Ohta-Nishinouchi Hospital, 2-5-20, Nishinouchi, Koriyama, Fukushima 963-8558, Japan; ^2^Department of Hematology, Ohta-Nishinouchi Hospital, 2-5-20, Nishinouchi, Koriyama, Fukushima 963-8558, Japan; ^3^Department of Internal Medicine, Ohta-Nishinouchi Hospital, 2-5-20, Nishinouchi, Koriyama, Fukushima 963-8558, Japan

## Abstract

Systemic lupus erythematosus (SLE) is an autoimmune disease that leads to a wide spectrum of clinical and immunological abnormalities. Hematologic abnormalities are an important manifestation of SLE. The incidence of autoimmune hemolytic anemia (AIHA) has been reported in approximately 10% of patients with SLE. Among them, mixed-type AIHA, which is caused by warm autoantibodies and cold hemagglutinin, is relatively rarely reported. We report the case of a 72-year-old woman, who was admitted to our hospital due to shortness of breath, jaundice, and severe anemia, with SLE and antiphospholipid syndrome (APS) complicated by mixed-type AIHA. Laboratory data revealed severe hemolytic anemia (low hemoglobin, high indirect bilirubin, and high lactate dehydrogenase levels), low complement levels, and the presence of antinuclear antibodies and lupus anticoagulant. Imaging results revealed pleural effusion and pulmonary embolisms, and echocardiogram revealed high estimated right ventricular pressure. She was diagnosed with SLE and APS complicated by mixed-type AIHA based on positive direct antiglobulin and cold agglutinin tests (thermal amplitude ≥30°C). As mixed-type AIHA is a severe and chronic condition, she was administered potent treatments with immunosuppressants. However, because she was a carrier of human T-cell leukemia virus type-1, only a moderate amount of prednisolone was administered. She refused to take warfarin. Fortunately, her symptoms and laboratory abnormalities improved after prednisolone administration, and no relapse occurred after tapering the prednisolone dose. Although mixed-type AIHA is characterized by fewer clinical symptoms than cold agglutinin disease, hemolytic anemia is more severe and chronic. Therefore, it is important to confirm the presence of cold agglutinins, which are active at ≥30°C in patients with SLE and warm AIHA. In addition, it is important to consider that AIHA is associated with thromboembolism, and patients with lupus anticoagulant or anticardiolipin antibodies having a history of AIHA are at a high risk of developing thrombosis.

## 1. Introduction

Systemic lupus erythematosus (SLE) is an autoimmune disease that leads to a wide spectrum of clinical and immunological abnormalities [1, 2]. SLE is characterized by arthritis, glomerulonephritis, hematological abnormalities, and autoantibody production [1, 2]. Its pathogenesis is influenced by multiple factors, including environmental and genetic susceptibility factors [3]. Autoreactive B cells and interferons are a key in SLE pathogenesis. Furthermore, the two disease-defining and pervasive immune features of SLE are the breakdown of tolerance to nucleic acids and activation of the interferon (IFN) system. Therefore, the hallmark of SLE is the presence of antibodies against nucleic acids, particularly those against naked and nucleosome-associated double-stranded DNA and RNA/RNA-binding proteins, and an IFN-stimulated gene signature in blood and affected tissues [4]. Nucleic acids bound to SLE autoantibodies induce IFN production upon being internalized by plasmacytoid dendritic cells providing a pathogenic link [5]. This link elucidates the fundamental role of endosomal toll-like receptor-mediated sensing of nucleic acids in SLE. Pleiotropic effects of IFN on dendritic cells and B cell differentiation have also been elucidated [4]. A genome-wide association study mapped >90 loci associated with SLE susceptibility in the last decade, and several single nucleotide polymorphisms were found to act additively. In addition, rare monogenic forms of SLE have been reported. Among the 730 SLE-associated polymorphisms, 21 lead to amino acid change, 484 exist within gene coding regions, and the rest are intergenic, suggesting a significant effect on gene regulation instead of protein sequence. Most loci associated with a risk of SLE are located within or near gene encoding products that aid in the clearance of immune complex, lymphocyte signaling, and type I IFN signaling [6]. The main hematological manifestations of SLE include anemia, leukopenia, and/or thrombocytopenia [7]. Among them, autoimmune hemolytic anemia (AIHA) with reticulocytosis, an important hematological abnormality, is a key criterion for SLE classification [8]. In addition, antiphospholipid syndrome (APS) can develop in the absence of evidence of autoimmune diseases, or it can be secondary to autoimmune diseases such as SLE [9]. APS is a thromboinflammatory disease caused by circulating autoantibodies that recognize cell surface phospholipids and phospholipid-binding proteins. This can enhance the risk of thrombotic events, pregnancy morbidity, and other autoimmune and inflammatory complications [10].

AIHA is a relatively rare disorder, affecting 1–3 in 100,000 individuals per year with a prevalence ratio of 17 : 100,000. AIHA is caused by autoantibodies directed against self-red blood cells [11, 12]. Serologically, AIHA cases are divided into warm (65%), cold (29%: cold hemagglutinin disease; 1%: paroxysmal cold hemoglobinuria), and mixed-type (5%) AIHA groups [13]. Clinically, AIHA may be idiopathic/primary (50% of cases) or secondary to lymphoproliferative syndrome (20%), autoimmune disease (20%), infections, and tumors [14]. In particular, AIHA has been reported in up to 10% of patients with SLE [15, 16]. Although SLE may be the underlying cause of the cold hemagglutinin disease, the main antierythrocyte antibody in SLE is warm-type immunoglobulin G (IgG) [7, 13]. Therefore, SLE-associated AIHA requires a careful serological diagnosis. Furthermore, anticardiolipin (ACL) antibodies are associated with Coombs-positive hemolytic anemia in patients with SLE [17]. Thus, AIHA could be a marker for a subset of patients, who are more susceptible to APS, with SLE [18, 19].

Here, we described the case of a 72-year-old woman diagnosed with SLE and APS complicated by mixed-type AIHA based on a direct antiglobulin test for IgG, C3, and a cold antibody at a thermal amplitude of 30°C.

## 2. Case Presentation

**Figure 1:**
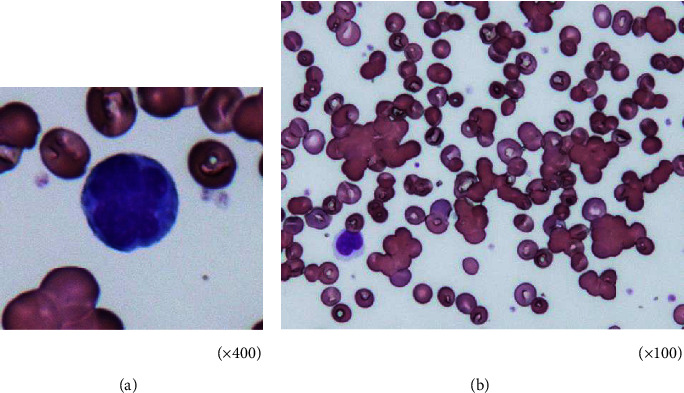
Peripheral blood smear examination showing flower cells (3.5% of leukocyte fraction) (a) and red blood cell agglutination (b).

**Figure 2:**
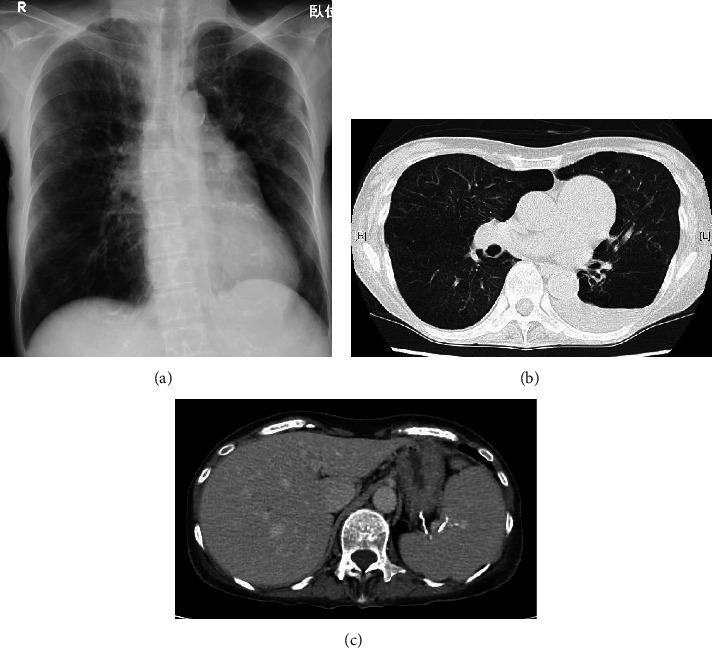
Chest X-ray revealing cardiac enlargement and emphysematous shadows in both the lungs (a). Computed tomography revealing emphysematous shadows in almost all lung fields, left-side pleural effusion (b), and splenomegaly (c).

**Figure 3:**
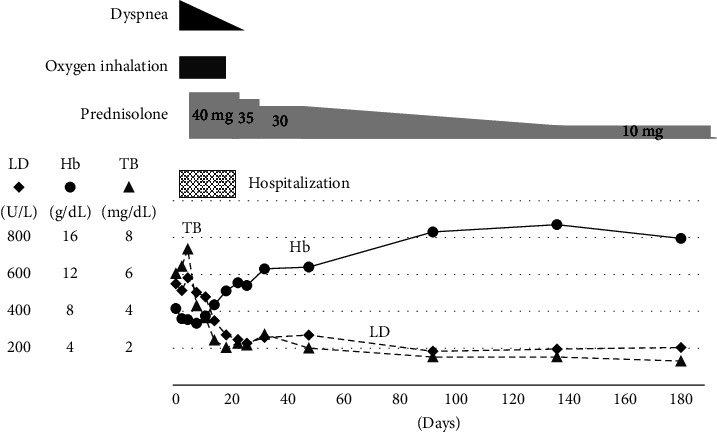
Clinical course. Hb: hemoglobin; LD: lactate dehydrogenase; TB: total bilirubin.

A 72-year-old woman was diagnosed twice (at 22 and 33 years of age) with hemolytic anemia and admitted to another hospital. Her medical condition improved after corticosteroid administration, and she was recommended to avoid cold conditions. Subsequently, her medical condition was stable without treatment; however, she experienced sun sensitivity and Raynaud's phenomenon in winters. She was admitted to our hospital due to dyspnea and severe anemia. She experienced progressive shortness of breath and weakness in the lower extremities 1 month before admission. She visited another clinic where laboratory tests revealed severe anemia and jaundice. There was no family history of thromboembolism. She had experienced an anaphylactic shock supposedly due to an antibiotic at 45 years of age and was diagnosed with chronic obstructive pulmonary disease at the age of 68 years. She had no history of thromboembolism and abortion. She had a history of smoking 20 cigarettes per day when she was aged 22–55 years, but she was not a habitual drinker. Her mother had rheumatoid arthritis and SLE. Her husband died of adult T-cell leukemia (ATL). Her daughter is a human T-cell leukemia virus type-1 (HTLV-1) carrier. On admission, her height and body weight were 164 cm and 41 kg, respectively. Her body temperature, blood pressure, pulse rate, and oxygen saturation were 36.8°C, 108/59 mmHg, 78 beats/min, and 92% under room air, respectively. Her eyelid conjunctiva was anemic and ocular conjunctiva was jaundiced. No heart murmur or abnormal chest sounds were heard on auscultation, and no edema was observed in her extremities. The laboratory findings revealed a red blood cell count of 1.3 × 10^6^/*μ*L, hemoglobin level of 7.2 g/dL, hematocrit level of 18.4%, total bilirubin level of 6.44 mg/dL, indirect bilirubin level of 5.98 mg/dL, lactate dehydrogenase level of 513 U/L, antinuclear antibody titer of 1 : 640 (speckled and cytoplasmic pattern), cold agglutinin titer of 1 : 512, and positivity for cryoglobulin and direct and indirect Coombs tests. Antibodies against hepatitis C virus and HTLV-1 were also detected. The direct antiglobulin test (DAT) revealed positivity for IgG and complement components (Table 1). Peripheral blood smear examination revealed flower cells (3.5% of leukocyte fraction) and red blood cell agglutination (Figure 1). Chest X-ray revealed cardiac enlargement and emphysematous shadows in both the lungs, and computed tomography revealed emphysematous shadows in almost all lung fields, left-side pleural effusion, and splenomegaly (Figure 2). However, brain magnetic resonance imaging revealed no ischemic findings, and no thrombi were detected upon performing venous echocardiography of the lower extremities. Nevertheless, a ventilation/perfusion lung scan revealed several small wedge-shaped defects in the peripheral lung field. An echocardiogram revealed a high right ventricular pressure of 58.3 mmHg, suggesting overload in the right heart. The patient was diagnosed with AIHA based on splenomegaly, low hemoglobin level, increased reticulocyte count, high titer of indirect bilirubin and lactate dehydrogenase, and positive Coombs tests [13]. In addition, according to the European League Against Rheumatism/American College of Rheumatology classification criteria for SLE [8], she was diagnosed with SLE as she met the criteria of having an antinuclear antibody titer >1 : 80, autoimmune hemolysis (4), pleural effusion (4), lupus anticoagulant (2), and low C3 and C4 levels (4); her total score was 14, which satisfied the requirement for a total score ≥10 for SLE diagnosis. Furthermore, she was diagnosed with APS using the Sydney criteria for APS classification [20] based on the detection of vascular thrombosis (pulmonary thrombosis) and lupus anticoagulant (LAC) in the plasma twice at an interval ≥12 weeks. Furthermore, she was diagnosed as an HTLV-1 carrier because no ATL lesion was observed and only 3.5% of atypical lymphoma was present in the peripheral blood [21]. Subsequently, as both DAT (IgG and complements) and cold agglutinin test were positive, we evaluated the thermal amplitude to exclude the possibility of the presence of clinically insignificant polyclonal cold agglutinins. The thermal amplitude was found to be 30°C (2 times dilution) in this patient. Therefore, she was diagnosed with mixed-type AIHA based on a positive DAT for IgG and C3 and positive cold agglutinin test with a thermal amplitude ≥30°C [13]. She received oxygen on admission. After the diagnosis of SLE and mixed-type AIHA, she was administered prednisolone (40 mg/day) since day 7 of hospitalization. Her dyspnea and hemolytic findings gradually improved after treatment, and oxygen supplementation was discontinued on day 16 of hospitalization. She refused to take warfarin, which was proposed for the treatment of APS. She was discharged on day 26 of hospitalization. The prednisolone dose was tapered during the follow-up period. As she was an HTLV-1 carrier, no immunosuppressants were administered. Although the prednisolone dose was reduced to 10 mg/day, she maintained a good condition with improved right heart overload 6 months later (Figure 3).

## 3. Discussion

Herein, we described the case of a patient diagnosed with SLE and APS complicated by mixed-type AIHA who was successfully treated with a moderate dose of prednisolone. The incidence rate of mixed-type AIHA (DAT + for IgG and C3d, with the coexistence of warm autoantibodies and high-titer cold agglutinins) is generally reported to be 5–10% [13, 14, 22, 23]. However, Mayer et al. reported that of 2194 patients with warm antibodies, only 2 (<0.1%) developed both warm antibodies and cold agglutinins with the clinical evidence of hemolytic anemia [24]. Therefore, the incidence of mixed-type AIHA characterized by the combined presence of cold agglutinin with high thermal amplitude and warm antibodies may be lower than that reported previously. Socol et al. reported that mixed-type AIHA is commonly secondary to SLE and lymphoma, and Shulman et al. considered SLE as the most frequently associated disorder with AIHA [22, 25]. Sokol et al. [22] also revealed that although it is a possible complication associated with SLE, only few cases of mixed-type AIHA have been reported [26, 27]; thus, it is likely to remain undiagnosed. The study patient was diagnosed with hemolytic anemia at a young age and was treated with glucocorticoids. The exacerbation of hemolytic symptoms during winters and Raynaud's symptoms suggested that hemolytic anemia most likely occurred due to cold agglutinin disease because cold-induced hemolysis, Raynaud's phenomenon, and acrocyanosis are not typical features of mixed-type AIHA.

Mixed-type AIHA often responds well to steroid therapy, but it is a chronic disease. The course of this case differs from the typical course of mixed-type AIHA as the patient remained untreated for a long time before admission. Therefore, the combined pathology of SLE and AIHA due to warm autoantibodies may have appeared later in this case. To the best of our knowledge, no study has discussed the order of appearance of warm autoantibodies and cold agglutinins in mixed-type AIHA. Although both antibodies are detected during the diagnosis in most cases, the order of appearance of antibodies is an interesting topic to consider in the pathogenesis of mixed-type AIHA. In addition, despite being an HTLV-1 carrier, she did not develop ATL; hence, HTLV-1 is unlikely the cause of secondary AIHA in this case.

ACL antibodies have been associated with Coombs-positive hemolytic anemia in patients with SLE. Sthoeger et al. proposed a direct role of ACL antibodies in the pathogenesis of hemolytic anemia because they can act as anti-red blood cell autoantibodies in some patients with SLE [17]. Furthermore, elevated levels of ACL antibodies have been reported in patients with idiopathic AIHA, and they are related to AIHA pathogenesis [28]. Although ACL antibodies were not detected in this patient, her disease status was complicated by APS, as indicated by the positive results for LAC and pulmonary thrombosis. Increased thrombin generation has been reported in patients with LAC [29]. Pullarkat et al. revealed that the presence of LAC was significantly associated with the occurrence of venous thromboembolism in patients with AIHA [30]. A systematic review and meta-analysis by Ames et al. revealed a higher pooled prevalence of LAC in patients with AIHA and venous thrombosis than in those with AIHA and without venous thrombosis [31]. Therefore, the presence of LAC in this patient was considered to contribute to the modified pathogenesis of mixed-type AIHA. There was no exacerbation of APS after the patient refused to take warfarin. Thus, the treatment of mixed-type AIHA with glucocorticoids may have inhibited thrombus generation.

Recently, it has been recommended to prescribe immunosuppressants and avoid using steroids to treat SLE wherever possible [32]. In this case, we considered using immunosuppressants along with glucocorticoids; however, as the patient was an HTLV-1 carrier, the administration of immunosuppressant was discontinued to avoid the possibility of developing ATL. Although hemolytic anemia improved with glucocorticoid therapy alone, future treatment management for the patient should be carefully decided considering she suffers from osteoporosis.

In conclusion, we reported the case of a patient diagnosed with SLE and APS complicated by mixed-type AIHA and successfully treated with a moderate dose of glucocorticoids. It can be concluded that despite the prevalence of warm autoimmune hemolysis in patients with SLE and AIHA, only a few patients had cold agglutinins with a thermal amplitude ≥30°C (mixed-type AIHA). Although mixed-type AIHA is characterized by fewer clinical symptoms than cold agglutinin disease, hemolytic anemia is more severe and chronic. Therefore, it is important to confirm the presence of cold agglutinins, which are active at ≥30°C in patients with SLE and warm AIHA. In addition, it is important to consider that AIHA is associated with thromboembolism, and patients with LAC or ACL antibodies having an AIHA background are at a higher risk of developing thrombosis. Considering the lack of a detailed understanding of the clinical course and pathogenesis of mixed-type AIHA, further research is warranted.

## Figures and Tables

**Table 1:** Laboratory findings on admission.

Peripheral blood	
White blood cell (/*μ*L)	8,000 (3,300–8,600)
Neutrophil (%)	58.6
Eosinophil (%)	1.0
Monocytes (%)	5.2
Lymphocytes (%)	33.2
Red blood cell (×10^6^/*μ*L)	1.3 (3.86–4.92)
Hemoglobin (g/dL)	7.2 (11.6–14.8)
Hematocrit (%)	18.4 (35.1–44.4)
Platelet (×10^3^/*μ*L)	240 (158–348)
Reticulocytes (%)	298.1 (3–22)
Coagulation study	
Prothrombin time (%)	84.3 (>70)
APTT (second)	84.8 (24–37)
Fibrinogen (mg/dL)	219 (150–400)
FDP (*μ*g/mL)	3.4 (<5)
D-dimer (*μ*g/mL)	1.4 (<1)
Blood chemistry	
Total protein (g/dL)	6.7 (6.6–8.1)
Albumin (g/dL)	3.2 (4.1–5.1)
Total bilirubin (mg/dL)	6.44 (0.4–1.5)
Direct bilirubin (mg/dL)	0.46 (0–0.3)
Indirect bilirubin (mg/dL)	5.98 (<0.9)
AST (U/L)	32 (13–30)
ALT (U/L)	7 (7–23)
LD (U/L)	513 (124–222)
ALP (U/L)	78 (38–113)
*γ*-GTP (U/L)	16 (9–32)
Blood urea nitrogen (mg/dL)	21.6 (8–20)
Creatinine (mg/dL)	0.42 (0.46–0.79)
Uric acid (mg/dL)	5.9 (2.6–5.5)
Na (mmol/L)	145 (138–145)
K (mmol/L)	3.3 (3.6–4.8)
Cl (mmol/L)	110 (101–108)
Ca (mg/dL)	8.3 (8.8–10.1)
Fe (*μ*g/dL)	198 (40–188)
UIBC (*μ*g/dL)	23 (155–350)
TIBC (*μ*g/dL)	221 (250–410)
Ferritin (ng/mL)	218.8 (4.6–204)
TSH (*μ*IU/mL)	1.39 (0.35–4.94)
Free-T4 (ng/dL)	1.1 (0.7–1.48)
Vitamin B12 (pg/mL)	375 (233–914)
Folic acid (ng/mL)	3.8 (3.6–12.9)
sIL-2R (U/mL)	816 (127–582)
BNP (pg/mL)	143.1 (<18.4)
KL-6 (U/mL)	583 (<500)
CRP (mg/dl)	0.11 (0–0.14)
Serological study	
IgG (mg/dL)	1,725 (861–1,747)
IgA (mg/dL)	424 (93–393)
IgM (mg/dL)	391 (50–269)
C3 (mg/dL)	25.9 (73–138)
C4 (mg/dL)	1.9 (11–31)
Rheumatoid factor (IU/mL)	113 (<15)
Cold agglutinin (fold)	512 (<64)
Cryoglobulin	+
Antinuclear Ab (fold)	640 (<40)
Speckled cytoplasmic	
Anti-ds-DNA Ab (IU/mL)	1.7 (<12)
Anti-Sm Ab	−
Anti-RNP Ab	−
Anti-SS-A Ab	−
Anti-SS-B Ab	−
Anti-CL*β*2GPI Ab (U/mL)	2.1 (<3.5)
Lupus anticoagulants	2.59 (<1.2)
HBV Ag	−
HCV Ab	+
HTLV-1 Ab	+
Electrophoresis	
Serum M-protein	−
Urine M-protein	−
Test for hemolysis	
Direct Coombs test	+
Direct antiglobulin test	
IgG	3+
Complements	3+
Indirect Coombs test	+
Donath–Landsteiner test	−
Urinalysis	
Protein	±
Occult blood	+

APTT, activated partial thromboplastin time; FDP, fibrin-fibrinogen degradation product; AST, aspartate aminotransferase; ALT, alanine aminotransferase; LD, lactate dehydrogenase; ALP, alkaline phosphatase; *γ*-GTP, *γ*-glutamyl transpeptidase; Na, sodium; K, potassium; Cl, chloride; Ca, calcium; Fe, iron; UIBC, unsaturated iron-binding capacity; TIBC, total iron-binding capacity; TSH, thyroid-stimulating hormone; T4, thyroxine; sIL-2R, soluble interleukin-2 receptor; BNP, brain natriuretic peptide; CRP, C-reactive protein; Ig, immunoglobulin; C3, third component of complement; C4, fourth component of complement; Ab, antibodies; CL*β*2GP1, cardiolipin *β*2 glycoprotein I; HBV, hepatitis B virus; HCV, hepatitis C virus; HTLV-1, human T-cell leukemia virus type-1. ^*∗*^Numbers in parentheses indicate reference range.

## Data Availability

No underlying data were collected or produced in this study.
